# Systematic identification of endogenous strong constitutive promoters from the diazotrophic rhizosphere bacterium *Pseudomonas stutzeri* DSM4166 to improve its nitrogenase activity

**DOI:** 10.1186/s12934-023-02085-3

**Published:** 2023-05-03

**Authors:** Guangle Yu, Xiaochen Li, Qiuyue Duan, Jun Fu, Youming Zhang, Hailong Wang, Ji Luan

**Affiliations:** State Key Laboratory of Microbial Technology, Institute of Microbial Technology, Helmholtz International Lab for Anti-infectives, Shandong University–Helmholtz Institute of Biotechnology, Shandong University, Binhai Rd 72, Qingdao, Shandong 266237 China

**Keywords:** Promoters, Biological nitrogen fixation, *Pseudomonas stutzeri*, RNA-seq, Luciferase assay, Metabolic engineering

## Abstract

**Background:**

Biological nitrogen fixation converting atmospheric dinitrogen to ammonia is an important way to provide nitrogen for plants. *Pseudomonas stutzeri* DSM4166 is a diazotrophic Gram-negative bacterium isolated from the rhizosphere of cereal *Sorghum nutans*. Endogenous constitutive promoters are important for engineering of the nitrogen fixation pathway, however, they have not been systematically characterized in DSM4166.

**Results:**

Twenty-six candidate promoters were identified from DSM4166 by RNA-seq analysis. These 26 promoters were cloned and characterized using the *firefly* luciferase gene. The strengths of nineteen promoters varied from 100 to 959% of the strength of the gentamicin resistance gene promoter. The strongest P12445 promoter was used to overexpress the biological nitrogen fixation pathway-specific positive regulator gene *nifA*. The transcription level of nitrogen fixation genes in DSM4166 were significantly increased and the nitrogenase activity was enhanced by 4.1 folds determined by the acetylene reduction method. The *nifA* overexpressed strain produced 359.1 µM of extracellular ammonium which was 25.6 times higher than that produced by the wild-type strain.

**Conclusions:**

The endogenous strong constitutive promoters identified in this study will facilitate development of DSM4166 as a microbial cell factory for nitrogen fixation and production of other useful compounds.

**Supplementary Information:**

The online version contains supplementary material available at 10.1186/s12934-023-02085-3.

## Background

Biological nitrogen fixation (*nif*) plays an important role in the global nitrogen cycle [1, 2]. It converts atmospheric dinitrogen to ammonia under microaerobic and nitrogen-free condition [3, 4] which can be used by plants for growth and development. Naturally organisms capable of fixing nitrogen are all prokaryotes including bacteria and archaea [5]. Legumes obtain most of their nitrogen from rhizobia bacteria residing in root nodules, however, many crop plants including cereals are nonleguminous and cannot form symbiotic associations [6].To meet the demand of improving crop yield, chemically synthesized nitrogen fertilizers have been widely applied in the world. Unfortunately, overuse of these chemical fertilizers has raised serious environmental problems [7–9]. Engineering of biological nitrogen fixation capacity in bacteria associating with non-legume crops will help to improve agricultural sustainability by reducing the dependence on chemically fertilizers [10–13].

*Nif* genes including the nitrogenase genes, the cofactor biosynthesis genes and electron transporter genes are organized in clusters on the chromosome of bacteria [14, 15]. The biological reduction of dinitrogen to ammonia are catalyzed by nitrogenases which are complex metalloenzymes [16, 17]. There are three classes of nitrogenases: the Mo-Nitrogenase, the V-Nitrogenase and the Fe-Nitrogenase, among which the Mo-containing nitrogenase is the most prevalent and the best characterized [18]. Mo-Nitrogenase is composed of two oxygen-sensitive components, the MoFe protein and the Fe protein. MoFe protein encoded by *nifD* and *nifK* is a heterotetramer which provides the active site for substrate reduction. Fe protein encoded by *nifH* is a homodimer which serves as an electron donor to the MoFe protein during catalysis [19–22]. Expression of *nif* genes is tightly regulated in diazotrophic bacteria by the pathway specific positive regulatory protein NifA [23] which belongs to bacterial enhancer-binding proteins (bEBPs) containing the AAA+ (ATPases associated with various cellular activities) domain [24, 25]. NifA protein binds to the specific sequence upstream of promoters of *nif* genes to activate their transcription. Transcription of *nifA* is regulated by the NtrB-NtrC two-component system in response to nitrogen source in diazotrophic proteobacteria such as *Klebsiella pneumoniae*, the FixL-FixJ two-component system in response to oxygen in symbiotic diazotrophs such as *Sinorhizobium meliloti*, or the RegS-RegR two-component system in response to redox in symbiotic diazotrophs such as *Bradyrhizobium japonicum* [6]. The activity of the NifA protein is modulated by the anti-activator protein NifL which is in response to oxygen and fixed nitrogen through interaction with GlnK in γ-Proteobacteria [12, 26–29]. In symbiotic diazotrophs that lack NifL, the domain structure of NifA proteins is different to those subject to NifL inhibition, and the activity of these NifA proteins is directly modulated by the oxidation state of the cell [30].

*Pseudomonas stutzeri* DSM4166 is a diazotrophic isolate from the rhizosphere of a “high fixing” *Sorghum nutans* cultivar [31]. Its *nif* gene cluster is 49 kb and contains 58 genes [32]. DSM4166 has the potential to be used as nitrogen fertilizer, therefore it is vital to improve its nitrogen fixation capacity. The nitrogenase activity of *Rhodobacter capsulatus* was enhanced by two folds through overexpressing the *rnf* electron transporter genes [33]. Overexpression of the NifH nitrogenase structural protein increased the nitrogen-fixing efficiency of *Azotobacter vinelandii* by two folds [34]. Increasing the copy number of the *nif* gene cluster in the cyanobacterium *Synechocystis* enhanced its nitrogen fixation activity by three folds [35]. The ammonia excretion capacity of *Azotobacter vinelandii* were enhanced through genetic manipulation by deleting the ammonium transporter AmtB [36], or introducing mutations in the NifA protein [37], the NifL protein [12, 38], or the glutamine synthetase [39]. The nitrogen fixation and the ammonia excretion capacities of *Pseudomonas stutzeri* A1501 were increased by overexpressing NifA and deleting the ammonium transporter AmtB [10, 13]. Novel noncoding RNAs involved in post-transcriptional regulation of nitrogenase expression were identified and characterized in *Pseudomonas stutzeri* A1501 [40–42]. Engineering of DSM4166 to increase its nitrogen fixation capacity needs promoters with different strengths, however, both exogenous and endogenous promoters have not been systematically characterized in DSM4166.

In this study, we characterized 26 candidate promoters identified by RNA-seq analysis of DSM4166 cultured in the LB rich medium and the PMM minimum medium. Ten promoters were stronger than the promoter of gentamicin resistance gene [43]. When we used the endogenous strong constitutive promoters to overexpress the nitrogen fixation pathway specific positive regulator gene *nifA*, the nitrogen fixation efficiency of DSM4166 were increased by 25.6 folds determined by the production of extracellular ammonium.

## Results and discussion

### Screening of strong constitutive promoters in the ***P. stutzeri*** DSM4166 transcriptome by RNA-seq

The LB rich medium and the PMM minimal medium were used for transcription analysis of all 4427 genes in DSM4166 (GenBank accession number: NC_017532.1). The cells in the mid-exponential phase and the early stationary phase were used for RNA-seq analysis (Fig. S1). Expression levels of genes from each sample were ranked from the highest to the lowest based on their values of read counts (Table S1). In the four samples (two time points in two media), there were 39 genes in the 3% cutoff, which means that 39 genes were in the top 3% of the most highly expressed genes under all culturing conditions (Fig. S2). Among these 39 genes, 13 genes with very short promoter regions (shorter than 100 bp) were excluded. Therefore, we chose 26 highly expressed genes with their corresponding promoter regions (Table 1) for further characterization.

**Table 1:** Selected 26 promoter regions from *P. stutzeri* DSM4166

Promoter ID	Locus Tag	Gene ID	CDS product
P22180	—	PSTAA_RS22180	Transfer-messenger RNA
P17270	PSTAA_3470	PSTAA_RS17270	30 S ribosomal protein S15
P19520	PSTAA_3915	PSTAA_RS19520	hypothetical protein
P06545	PSTAA_1295	PSTAA_RS06545	Cold-shock protein
P03385	PSTAA_0660	PSTAA_RS03385	PrkA family serine protein kinase
P10035	PSTAA_1994	PSTAA_RS10035	DUF1722 domain-containing protein
P19680	PSTAA_3946	PSTAA_RS19680	Pyruvate dehydrogenase
P03670	PSTAA_0716	PSTAA_RS03670	DNA-directed RNA polymerase subunit beta
P20930	PSTAA_4197	PSTAA_RS20930	TAXI family TRAP transporter solute-binding subunit
P04125	PSTAA_0809	PSTAA_RS04125	Indolepyruvate ferredoxin oxidoreductase
P12415	PSTAA_2481	PSTAA_RS12415	PA2779 family protein
P07800	PSTAA_1544	PSTAA_RS07800	OmpA family protein
P09555	PSTAA_1901	PSTAA_RS09555	2-oxoglutarate dehydrogenase E1 component
P10530	PSTAA_2094	PSTAA_RS10530	Endopeptidase La
P09495	PSTAA_1888	PSTAA_RS09495	Flagellin
P12505	PSTAA_2502	PSTAA_RS12505	NAD-glutamate dehydrogenase
P16030	PSTAA_3217	PSTAA_RS16030	Cold-shock protein
P05750	PSTAA_1138	PSTAA_RS05750	Amino acid ABC transporter substrate-binding protein
P10410	PSTAA_2070	PSTAA_RS10410	Phosphoenolpyruvate synthase
P02720	PSTAA_0536	PSTAA_RS02720	D-amino acid dehydrogenase
P11845	PSTAA_2363	PSTAA_RS11845	30 S ribosomal protein S1
P07835	PSTAA_1551	PSTAA_RS07835	Membrane protein
P18460	PSTAA_3701	PSTAA_RS18460	NADP-dependent isocitrate dehydrogenase
P18595	PSTAA_3729	PSTAA_RS18595	DUF1631 domain-containing protein
P11670	PSTAA_2329	PSTAA_RS11670	NADP-dependent isocitrate dehydrogenase
P12445	PSTAA_2489	PSTAA_RS12445	Outer membrane porin, OprD family

### Characterization of chosen promoters using the ***firefly*** luciferase reporter gene

**Fig. 1:**
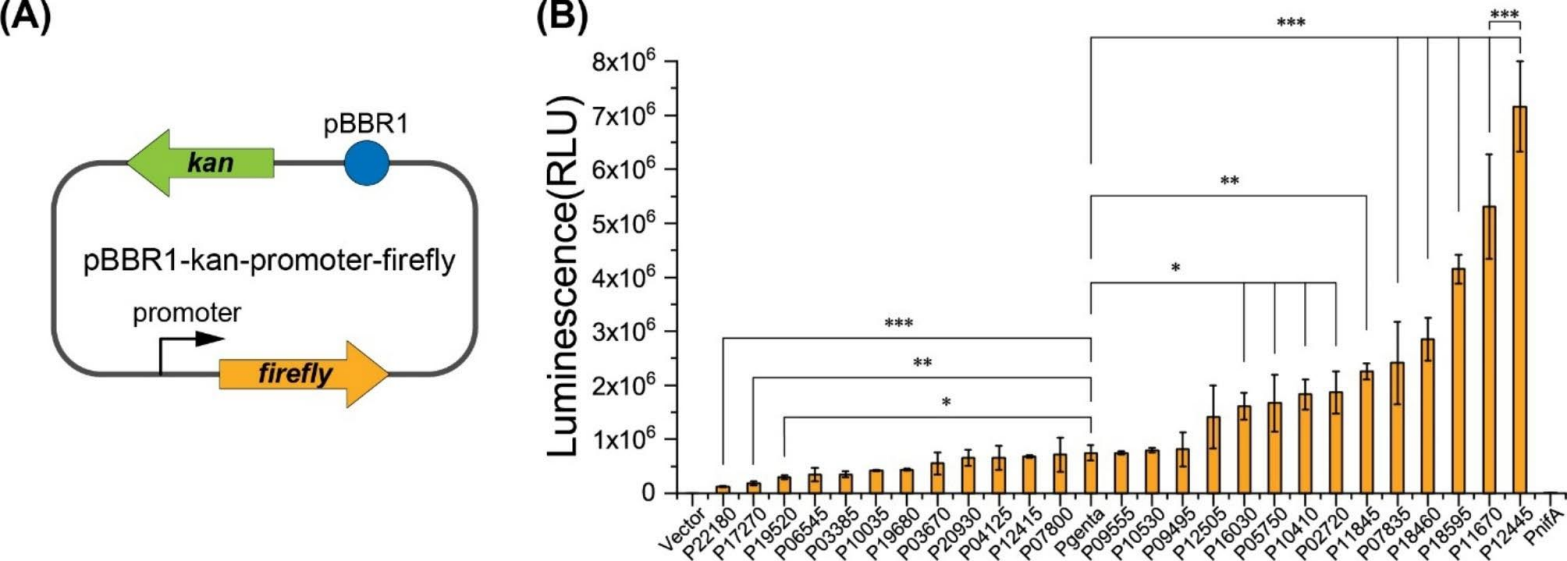
Luciferase assay of constitutive promoters in *P. stutzeri* DSM4166 in the LB medium under the aerobic condition. (**A**) Schematic of the plasmid containing different promoters. pBBR1: replicon; *kan*: kanamycin resistance gene; *firefly*: *firefly* luciferase reporter gene. **(B)** Luciferase activity of plasmids containing different promoters in DSM4166. Vector: the promoterless plasmid. P_genta_: the promoter of the gentamicin resistance gene. P_nifA_: the promoter of the nitrogen fixation pathway specific positive regulator gene *nifA*. Error bars indicate the standard deviations of three replicates (*n* = 3). The P-value cutoff for all the plots is 0.05. * P < 0.05, ** P < 0.01, and *** P < 0.001. One-way ANOVA test with Tukey Pairwise comparisons was used to compute statistical significance

Each promoter region that we selected to clone is the intergenic region between the highly expressed gene and its upstream gene. Sequences of these promoters were listed in Table S3. Each promoter and the *firefly* luciferase gene were assembled into a pBBR1 vector (Fig. 1A) by recombineering [44]. The promoter of the gentamicin resistance gene (P_genta_) was used as the control because the gentamicin resistance gene is a good selection marker in DSM4166. The verified plasmids were electroporated into DSM4166 for the luciferase activity assay in the LB medium under the aerobic (air) condition. As shown in Fig. 1B, activities of these promoters varied from 17 to 959% of that of P_genta_. Thirteen promoters had the same strength as P_genta_. Ten promoters were stronger than P_genta_. P12445 was the strongest among them.

**Fig. 2:**
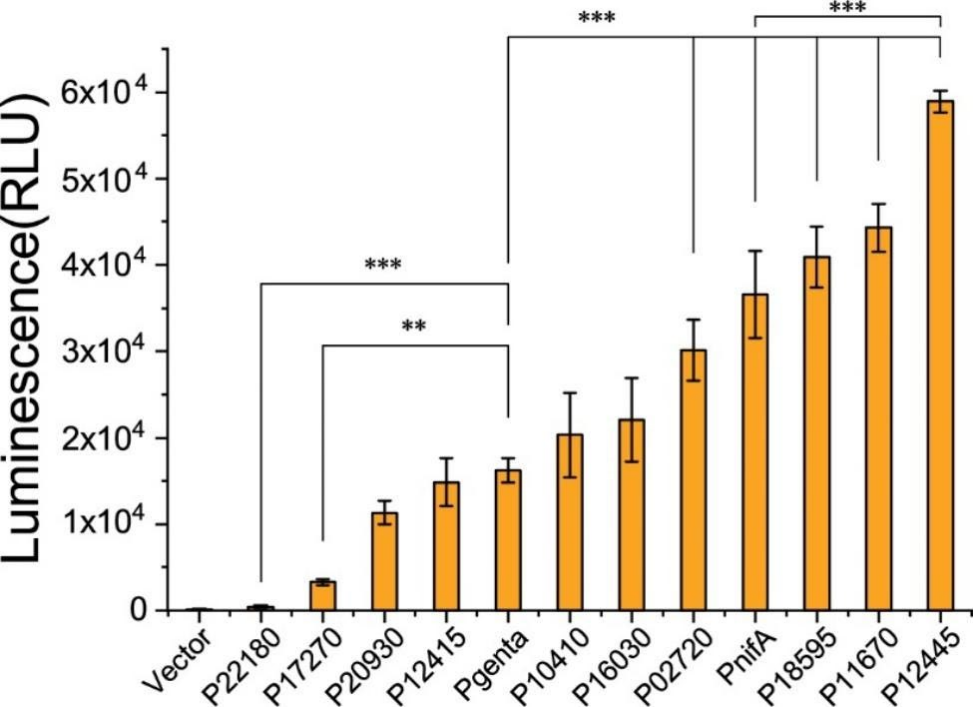
Luciferase activity of plasmids containing different promoters in DSM4166 in the nitrogen free medium K under the under the microaerobic (1% oxygen) condition. Vector: the promoterless plasmid. P_genta_: the promoter of the gentamicin resistance gene. P_nifA_: the promoter of the nitrogen fixation pathway specific positive regulator gene *nifA*. Error bars indicate the standard deviations of three replicates (*n* = 3). The P-value cutoff for all the plots is 0.05. * P < 0.05, ** P < 0.01, and *** P < 0.001. One-way ANOVA test with Tukey Pairwise comparisons was used to compute statistical significance

DSM4166 strains harboring the pBBR1-kan-firefly plasmids carrying promoters weaker than P_genta_ (P22180 and P17270), promoters having the same strength as P_genta_ (P20930, P12415), and promoters stronger than P_genta_ (P16030, P10410, P02720, P18595, P11670, and P12445) in the LB medium under the aerobic condition were then cultivated under the nitrogen fixation condition in which the nitrogen-free medium K [41] and the microaerobic (1% oxygen) condition was used for the luciferase activity assay. As shown in Fig. 2, activities of these promoters under the nitrogen fixation condition showed almost the same pattern as that in the LB medium under the aerobic condition. P22180 was the weakest and P12445 was the strongest. P02720, P18595, P11670, and P12445 were significantly stronger than P_genta_. P12445 was significantly stronger than P_nifA_.

### Enhancing the nitrogen fixation activity of DSM4166 using endogenous strong constitutive promoters

As nitrogenases are highly sensitive to oxygen, the nitrogenase activity of the wild-type DSM4166 strain cultivated in the nitrogen-free medium K was detected at a range of oxygen concentrations (0.5%, 1%, 2%, and 4%) by the acetylene reduction method [41]. As shown in Fig. S3, the oxygen concentration for optimal nitrogenase activity of DSM4166 is 0.5% or 1%. The nitrogenase activity of DSM4166 at 0.5% oxygen (278 nmol ethylene h ^− 1^ (mg protein) ^−1^) is almost the same as that at 1% oxygen (287 nmol ethylene h ^− 1^ (mg protein) ^−1^). The nitrogenase activity at 2% oxygen is very low and it cannot be detected at 4% oxygen. Therefore, we used 1% oxygen for the nitrogenase activity assay in the following experiments.

Because P_nifA_ is significantly weaker than P12445 under the nitrogen fixation condition (Fig. 2), the nitrogenase activity of DSM4166 could be improved if the nitrogen fixation pathway specific positive regulator NifA is overexpressed by the stronger P12445 promoter. The P11670 promoter was also selected to overexpress the NifA protein because of its high transcriptional activity under the nitrogen fixation condition. To facilitate evaluating expression levels of different DNA constructs at an identical locus in DSM4166, the *phiC31 attB* site was inserted at the PSTAA_RS19520 site (GenBank accession number: NC_017532.1) in the chromosome by single crossover to generate the DSM4166-attB recombinant strain (Fig. S4A). The PSTAA_RS19520 gene encodes a hypothetical protein and insertion of the *phiC31 attB* site did not affect the nitrogenase activity of DSM4166 (Fig. S4B). All DNA constructs can be integrated at the same *attB* site in the DSM4166 chromosome via site-specific recombination mediated by the PhiC31 integrase. The *nifA* gene was cloned downstream of P11670 and P12445 in the p15A-oriT-phiC31 plasmid (Fig. S4C) and then the P11670-nifA and P12445-nifA cassettes were respectively integrated at the *phiC31 attB* site on the chromosome of the DSM4166-attB strain to obtain recombinant strains DSM4166-P11670-nifA and DSM4166-P12445-nifA.

**Fig. 3:**
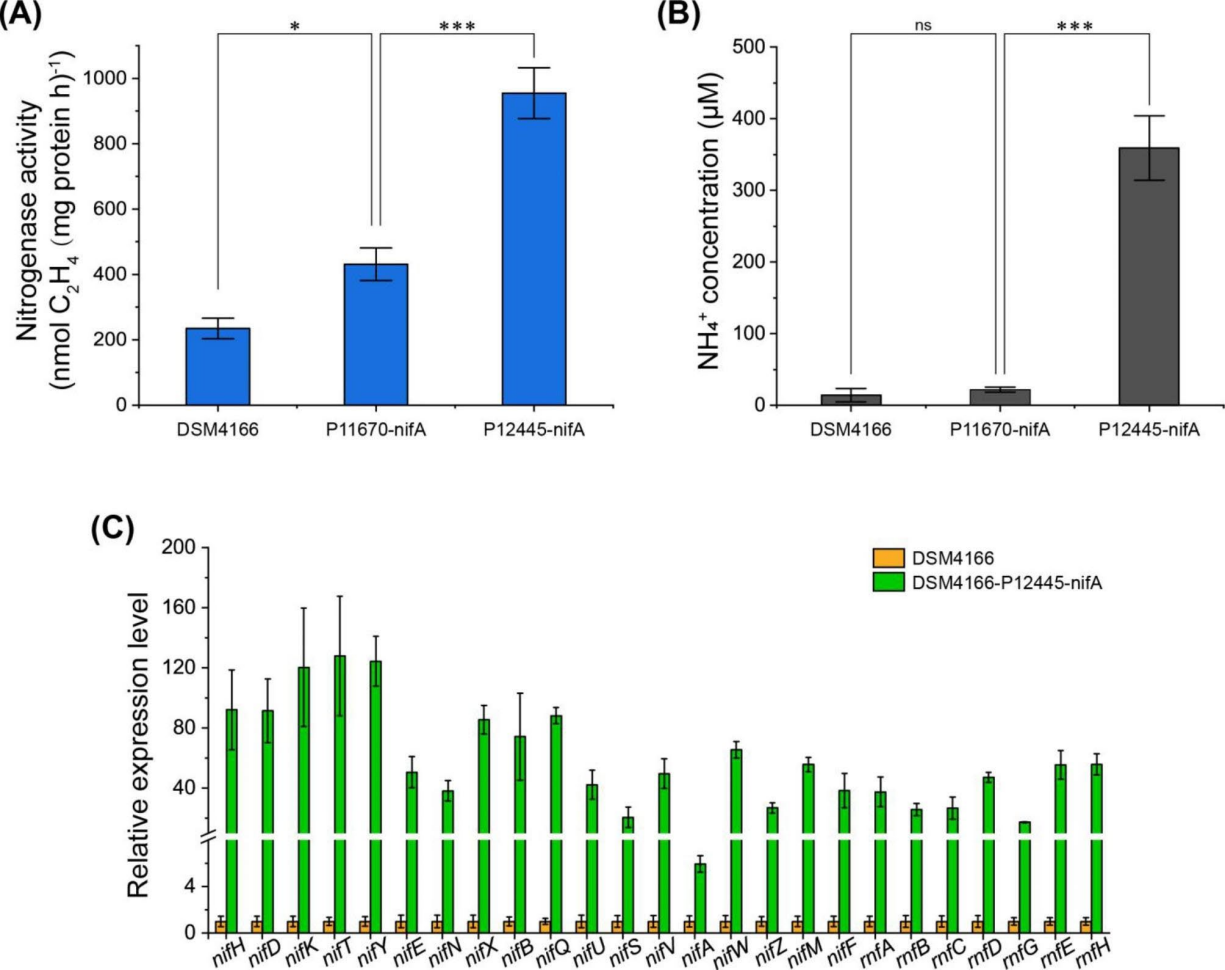
Enhanced nitrogenase activity by overexpressing *nifA* using endogenous strong constitutive promoters in DSM4166. (**A**) The nitrogenase activity of DSM4166 and *nifA* overexpression strains cultivated in the nitrogen-free medium K at 1% oxygen determined by the acetylene reduction method. (**B**) The concentration of extracellular ammonium produced by DSM4166 and *nifA* overexpression strains. (**C**) The transcriptional level of selected 25 *nif* genes in the P12445-nifA overexpression strain relative to DSM4166 cultivated in the same condition as A. Error bars indicate the standard deviations of three replicates (*n* = 3). The P-value cutoff for all the plots is 0.05. * P < 0.05, ** P < 0.01, and *** P < 0.001. ns, not significant. One-way ANOVA test with Tukey Pairwise comparisons was used to compute statistical significance

The nitrogenase activities of DSM4166-P11670-nifA and DSM4166-P12445-nifA under the nitrogen fixation condition determined by the acetylene reduction method were increased by 1.8 and 4.1 folds compared with the wild-type DSM4166 respectively (Fig. 3A). Quantitative real-time PCR (qRT-PCR) analysis results suggested that transcription levels of *nif* genes in the DSM4166-P12445-nifA strain were 25 ~ 127 times higher than that in the wild-type DSM4166 (Fig. 3C).

The ammonium secretion of the wild-type DSM4166, DSM4166-P11670-nifA, and DSM4166-P12445-nifA strains were determined by measuring the concentration of ammonium in the supernatant of cultures (Fig. 3B). The highest ammonium concentration (359.1 µM) was obtained in the supernatant of the DSM4166-P12445-nifA strain. The ammonium concentration of the DSM4166-P11670-nifA strain was 1.6 times higher than that of the wild-type DSM4166 strain and 16.3 times lower than that of the DSM4166-P12445-nifA strain. This result suggested that the surplus ammonium was secreted by DSM4166 strains. Secretion of the excess nitrogen fixed by DSM4166 strains will favor fertilizing crops or producing ammonium in the bioreactor. However, we did not test if the engineered DSM4166 strains would work in the rhizosphere or during plant-bacteria interaction.

**Fig. 4:**
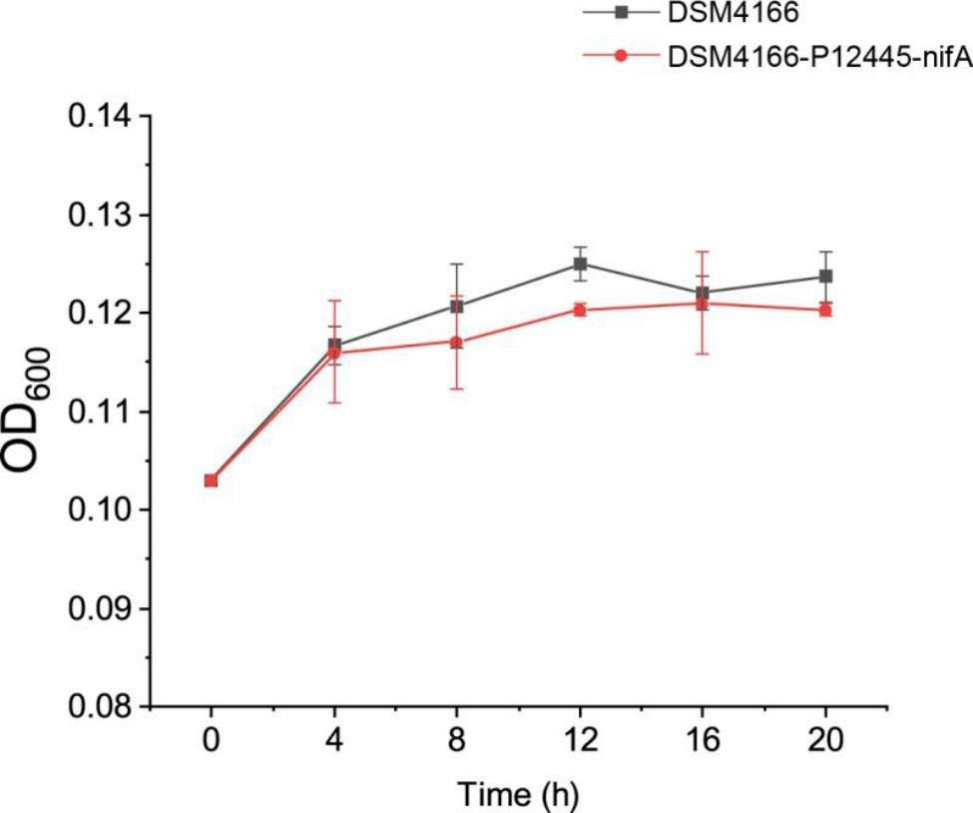
The growth curve of DSM4166 and *nifA* overexpression strains cultivated in the medium K at 99% N_2_ and 1% O_2_

An ammonium-excreting *P. stutzeri* A1501 engineered strain 1568/pVA3 constructed by overexpressing NifA and deleting the ammonium transporter AmtB was recently reported to produce 20.3 µM of extracellular ammonium after 72 h incubation [13]. In this study, the DSM4166-P12445-nifA strain produced 18 times more extracellular ammonium than the 1568/pVA3 strain with a shorter incubation (24 h). Furthermore, *nifA* overexpression under the P12445 promoter had little effect on the growth of DSM4166 in the medium K at 99% N_2_ and 1% O_2_ (Fig. 4).

## Conclusion

With the development of synthetic biology, promoters have been widely used for modulating gene expression to optimize biosynthetic pathways for the synthesis of important compounds. Twenty-three genes for biosynthesis of the insecticide spinosad and twenty-five genes for biosynthesis of the anticancer drug salinomycin had been refactored using multiple endogenous strong constitutive promoters to obtain artificial gene clusters with higher transcription level and higher compound productions [45, 46]. Gene cluster refactoring with strong constitutive promoters was also used to activate silent gene clusters for discovery and characterization of new natural products [47–49]. The nitrogen fixation gene cluster from *Klebsiella oxytoca* was refactored into artificial operons under the control of synthetic promoters, ribosome binding sites, and terminators to removes all native regulations [50]. The *nif* gene cluster refactoring also facilitated controlling the nitrogen fixation activity of bacteria under different growth conditions [51]. In this study, we identified a panel of endogenous strong constitutive promoters in *Pseudomonas stutzeri* strain DSM4166. The nitrogen fixation activity of DSM4166 was enhanced by overexpressing the nitrogen fixation pathway specific positive regulator using these promoters.

The Gram-negative soil bacterium *P. stutzeri* DSM4166 has the potential to be developed as a good microbial cell factory. Large DNA constructs can be easily transformed into this strain and integrated into its chromosome through transposition with high efficiency [52]. The *phiC31* site-specific recombination system was also established in this study. Its whole genome sequence had been published in 2011 and rhizosphere competence genes required in root colonization were identified such as denitrification and chemotaxis besides nitrogen fixation [32]. The endogenous constitutive promoters characterized in this study will facilitate engineering of *P. stutzeri* DSM4166 to further improve its nitrogen fixation capacity or produce useful chemicals.

## Methods

### Bacteria strains and culture conditions

*Escherichia coli* strains were cultured at 37 °C in LB medium. The auxotrophic conjugation donor strain *E. coli* WM3064 was maintained in the LB medium containing 1 mM of DL-α,ε-diaminopimelic acid [53]. *Pseudomonas stutzeri* DSM4166 strains were cultured at 30 °C in LB medium or PMM medium (8.0 g L^− 1^ K_2_HPO_4_·3H_2_O, 5.0 g L^− 1^ KH_2_PO_4_, 1.0 g L^− 1^ (NH_4_)_2_SO_4_, 6.6 g L^− 1^ sodium succinate, pH 7.0. Add 1.2 ml 1 M MgSO_4_ after autoclaving) under the aerobic condition or in the nitrogen-free medium K (0.4 g L^− 1^ KH_2_PO_4_, 0.1 g L^− 1^ K_2_HPO_4_, 0.1 g L^− 1^ NaCl, 0.2 g L^− 1^ MgSO_4_·7H_2_O, 0.01 g L^− 1^ MnSO_4_·H_2_O, 0.01 g L^− 1^ Fe_2_(SO_4_)_3_·H_2_O, 0.01 g L^− 1^ Na_2_MoO_4_·H_2_O, 10 mL L^− 1^ Sodium DL-lactate, pH 6.8) under microaerobic (nitrogen fixation) condition. Concentrations of antibiotics used in this study were: kanamycin, 15 µg mL^− 1^; apramycin, 20 µg mL^− 1^; gentamicin 2 µg mL^− 1^.

### ***Pseudomonas stutzeri*** cultivation and RNA-seq analysis

*P. stutzeri* DSM4166 culture was grown in the LB rich medium and the PMM minimal medium at 30 °C overnight with shaking at 950 rpm in an Aoheng thermomixer. Each 500 µL of overnight culture was inoculated into 50 mL of fresh LB medium or PMM medium in a 250-mL flask, and cultivated at 30 °C. The growth curve was determined by measuring OD_600_ of cell cultures every 2 h (Fig. S1). Cells were collected at 10 h (mid-exponential phase) and 14 h (early stationary phase) in LB medium, and 28 h (mid-exponential phase) and 38 h (early stationary phase) in PMM medium. Three replicates were set for each culturing condition. Twenty milliliters of culture from each replicate were mixed and centrifuged. The cell pellets frozen in dry ice were sent to Novogene Corporation (Beijing, China) for total RNA extraction and sequencing. The RNA integrity number (RIN) of samples was assessed using the RNA Nano 6000 Assay Kit of the Bioanalyzer 2100 system (Agilent Technologies, CA, USA). RIN of all samples were larger than 8.9 (Table S4) and recognized as good quality for library preparation. Samples were sequenced on an Illumina Hiseq 2500 platform and paired-end reads were generated. The *P. stutzeri* DSM4166 reference genome (GenBank accession number: NC_017532.1) was used for alignment. HTSeq v0.6.1 was used to count the reads numbers mapped to each gene (Table S1). Genes in each sample were ranked from the highest to the lowest expression using the values of read counts.

### DNA manipulation

The pBBR1-kan vector, the *firefly* reporter gene and promoters were amplified by PCR using the PrimeSTAR Max DNA Polymerase (Takara, cat. no. R045A) and oligonucleotides listed in Table S5. These three fragments were assembled by linear plus linear homologous recombination (LLHR) mediated by RecET in *E. coli* GB05-dir [44] to form the plasmid pBBR1-kan-promoter-firefly. After restriction analysis and sequencing, the correct plasmids were transformed into *P. stutzeri* DSM4166 by electroporation for luciferase assay.

For construction of the DSM4166-attB strain, the pK18mob vector [54] and the PSTAA_19520 segment were amplified by PCR using oligonucleotides listed in Table S6. These two fragments were assembled by LLHR in *E. coli* GB05-dir to form the plasmid pK18mob-19520-attB. After restriction analysis and sequencing, the correct pK18mob-19520-attB plasmid was transformed into *E. coli* WM3064 for conjugation. *P. stutzeri* DSM4166-attB exconjugants were screened by colony PCR using 19520-check-F/lac-seq and 19520-check-R/pK18-seq listed in Table S6.

For construction of DSM4166-P12445-nifA and DSM4166-P11670-nifA strains, the p15A vector, P12445, P11670, and the *nifA* gene were amplified by PCR using oligonucleotides listed in Table S7. Corresponding PCR products were assembled by LLHR in *E. coli* GB05-dir. After restriction analysis and sequencing, the correct plasmids were transfer into *E. coli* WM3064 for conjugation. *P. stutzeri* DSM4166 exconjugants were screened by colony PCR using 19520-check-F/nifA-5out and lac-seq/nifA-3out listed in Table S7.

### Luciferase assay

The Single-Luciferase (Firefly) Reporter Assay Kit (TransDetect, cat. no. FR101-01) was used to check the strength of the promoters. *P. stutzeri* DSM4166 cells harboring pBBR1-kan-promoter-firefly were inoculated into 1.0 mL of LB supplemented with kanamycin and incubated at 30 °C overnight with shaking at 950 rpm in an Eppendorf thermomixer. Cells were collected by centrifugation and washed once with phosphate buffer saline. Cells suspended in phosphate buffer saline with the OD_600_ = 1.0 were used to detect the fluorescence according to the manufacturer’s protocol.

For detecting the strength of the promoters in microaerobic (nitrogen fixation) condition, the overnight cultures of *P. stutzeri* DSM4166 cells harboring pBBR1-kan-promoter-firefly were collected and washed by nitrogen-free medium K. Then 1 mL of bacterial suspension (OD_600_ = 1.0) was added to a 100 mL anaerobic serum bottle which contains 9 mL of nitrogen-free medium K. The gas mixture (99% Ar + 1% O_2_) was blown into anaerobic serum bottle for 3 min to replace the headspace air, and the culture was incubated at 30 °C for 16 h with shaking at 200 rpm. Cells were collected by centrifugation and washed once with phosphate buffer saline. Cells suspended in phosphate buffer saline with the OD_600_ = 1.0 were used to detect the fluorescence according to the manufacturer’s protocol.

### Quantitative real-time PCR (qRT-PCR) analysis

The overnight cultures of the engineered *P. stutzeri* DSM4166 strains incubated in LB liquid medium were collected by centrifugation and washed with nitrogen-free medium K. Then 1 mL of diluted bacterial suspension (OD_600_ = 1.0) were added to a 100 mL anaerobic serum bottle which contains 9 mL of nitrogen-free medium K. The gas mixture (99% Ar + 1% O_2_) was blown into anaerobic serum bottle for 3 min to replace the headspace air, and the culture was incubated at 30 °C for 4 h with shaking at 200 rpm. After incubation, cells were collected by centrifugation. Total RNA was extracted using the RNAprep pure Kit (Tiangen, cat. no. DP430). DNA elimination and reverse transcription was performed with the PrimeScript RT reagent Kit with gDNA Eraser (Takara, cat. no. RR047A). The qRT-PCR was performed on StepOnePlus Real-Time PCR System (Applied Biosystems) using TB Green Premix Ex Taq (Takara, cat. no. RR420A) according to the manufacturer’s protocol. Oligonucleotides were listed in Table S8. The endogenous gene *gapdh*, encoding glyceraldehyde phosphate dehydrogenase, was used as the internal control.

### Nitrogenase activity assay

Nitrogenase activity was determined using the acetylene reduction assay [55]. *P. stutzeri* DSM4166 strains were incubated in LB liquid medium supplemented with appropriate antibiotics at 30 °C overnight with shaking. Cells were then collected by centrifugation and washed with nitrogen-free medium K, and resuspended to achieve an OD_600_ of 1.0 in medium K. Then 1 mL of diluted bacterial suspension were added to a 100 mL anaerobic serum bottle which contains 9 mL of nitrogen-free medium K. The gas mixture (99% Ar + 1% O_2_) was blown into anaerobic serum bottle for 3 min to replace the headspace air, 10% of acetylene gas (10 mL) was injected and the culture was incubated at 30 °C for 4 h with shaking at 200 rpm. Ethylene production was detected by Shimadzu GC2014 gas chromatograph equipped with a flame ionization detector (FID). We used a capillary column (KB-Al_2_O_3_/Na_2_SO_4_, 30-m length, 0.53-mm inner diameter, 20-µm film thickness; Shimadzu) with the following detection conditions: the injection port temperature was 100 °C, the transfer line temperature was 100 °C, the FID temperature was 180 °C. The carrier gas, ultra-high-purity nitrogen, flowed at a constant rate of 3 mL min^− 1^. One mL of gas samples was injected and the injections were split at a ratio of 1:40. The data was collected for 5 min. The ethylene gas was used as standard. After that, the cells were collected by centrifugation to determine the concentration of total proteins by the Bradford method [56] using bovine-serum albumin as standard. The nitrogenase activity was calculated by the following formula:

$$\frac{\frac{\text{Peak area of ethylene }\left(\text{experimental group}\right)}{\text{Peak area of ethylene }\left(\text{1 nmol}\right)}\times 100\left(\text{mL}\right)}{\text{Total protein content}\times \text{4h}}$$

### Quantification of ammonia concentration

To detect the concentration of ammonia excreted from engineered strains, cells from an overnight culture in LB medium supplemented with appropriate antibiotics were centrifuged and resuspended in 100 mL anaerobic serum bottle containing 10 ml nitrogen-free medium K at OD_600_ of 0.1. The gas mixture (99% N_2_ + 1% O_2_) was blown into anaerobic serum bottle for 3 min to replace the headspace air. After inoculation at 30 °C with shaking at 200 rpm for 24 h, the concentration of ammonium in the supernatant was measured using the Nessler’s reagent method [57]. Ammonium chloride was used as the standard.

## Electronic supplementary material

Below is the link to the electronic supplementary material.

Additional file 1: Figure S1. The growth curve of *P. stutzeri* DSM4166 in the LB medium and the PMM medium; Figure S2. Venn diagram of genes which were selected in 3% cut off according to gene expression level in RNA-seq; Figure S3. Schematic of construction of DSM4166-attB strain and plasmids of overexpressing *nifA* gene; Table S3. The sequence of screened endogenous promoters from *P. stutzeri* DSM4166. Table S4. Quality evaluation of samples for RNA-seq; Table S5. Oligonucleotides for cloning of screened endogenous promoter regions from *P. stutzeri* DSM4166; Table S6. Oligonucleotides used for construction of DSM4166-attB strain; Table S7. Oligonucleotides used for construction of DSM4166-P12445-nifA and DSM4166-P11670-nifA; Table S8. Oligonucleotides for quantitative real-time PCR; Table S9. Nitrogenase activity of DSM4166, DSM4166-P11670-nifA and DSM4166-P12445-nifA; Table S10. Quantitative real-time PCR analysis of *nif* genes in DSM4166 and DSM4166-P12445-nifA strains; Table S11. The concentration of extracellular ammonium produced by DSM4166 and *nifA* overexpression strains.

Additional file 2: Table S1. Read counts of RNA-seq samples.

Additional file 3: Table S2. The top 3% of the most highly expressed genes in the four samples.

## Data Availability

All data for this study are included in this published article and its additional file.
