# Supplementary material for: Systematic identification of endogenous strong constitutive promoters from the diazotrophic rhizosphere bacterium Pseudomonas stutzeri DSM4166 to improve its nitrogenase activity
Source: Microb Cell Fact. 2023 May 3;22:91. doi: 10.1186/s12934-023-02085-3 (PMC10155442; doi:10.1186/s12934-023-02085-3)
Supplement: Supplementary file 1 — Additional file 1: Figure S1. The growth curve of P. stutzeri DSM4166 in the LB medium and the PMM medium; Figure S2. Venn diagram of genes which were selected in 3% cut off according to gene expression level in RNA-seq; Figure S3. Schematic of construction of DSM4166-attB strain and plasmids of overexpressing nifA gene; Table S3. The sequence of screened endogenous promoters from P. stutzeri DSM4166. Table S4. Quality evaluation of samples for RNA-seq; Table S5. Oligonucleotides for cloning of screened endogenous promoter regions from P. stutzeri DSM4166; Table S6. Oligonucleotides used for construction of DSM4166-attB strain; Table S7. Oligonucleotides used for construction of DSM4166-P12445-nifA and DSM4166-P11670-nifA; Table S8. Oligonucleotides for quantitative real-time PCR; Table S9. Nitrogenase activity of DSM4166, DSM4166-P11670-nifA and DSM4166-P12445-nifA; Table S10. Quantitative real-time PCR analysis of nif genes in DSM4166 and DSM4166-P12445-nifA strains; Table S11. The concentration of extracellular ammonium produced by DSM4166 and nifA overexpression strains. [file 12934_2023_2085_MOESM1_ESM.pdf]

Supplemental Information

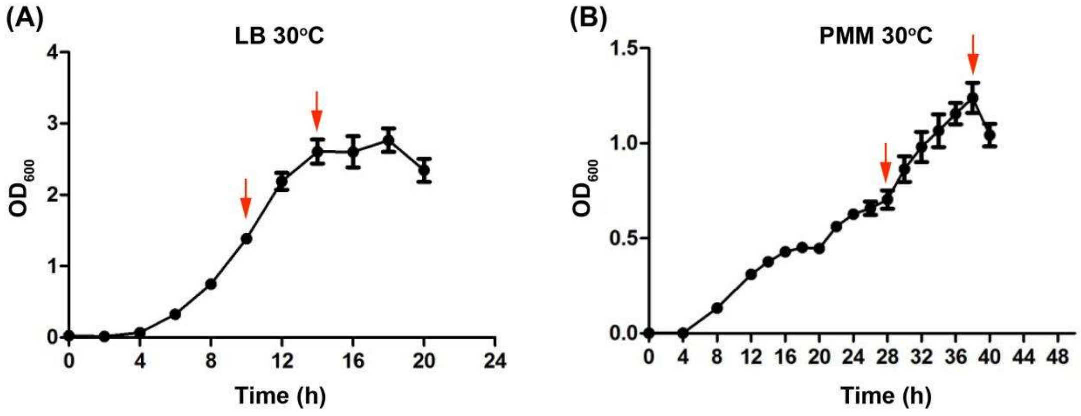

Fig. S1. The growth curve of *P. stutzeri* DSM4166 in the LB medium and the PMM medium. For each culture condition, two time point (indicated by the red arrow) were selected for RNA-seq analysis according the growth curve.

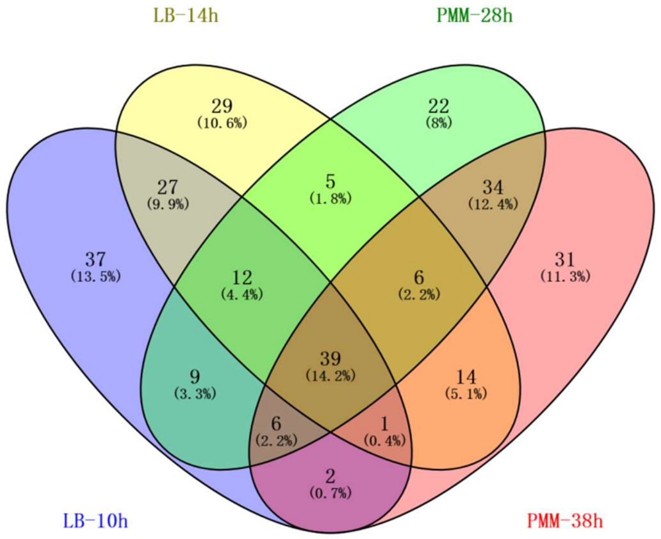

Fig. S2. Venn diagram of genes which were selected in 3% cut off according to gene expression level in RNA-seq. Expression levels of genes from each sample were ranked from the highest to the lowest based on their values of read counts. There were 133 genes in the 3% cutoff in the four samples (Table S2). Venn diagram was drawn according to Table S2.

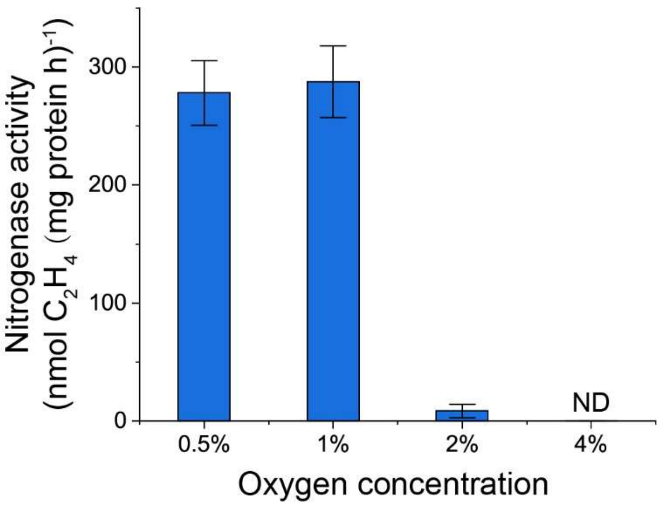

Fig. S3. The nitrogenase activity of the wild-type DSM4166 strain cultivated in the nitrogen-free medium K at different oxygen concentrations.

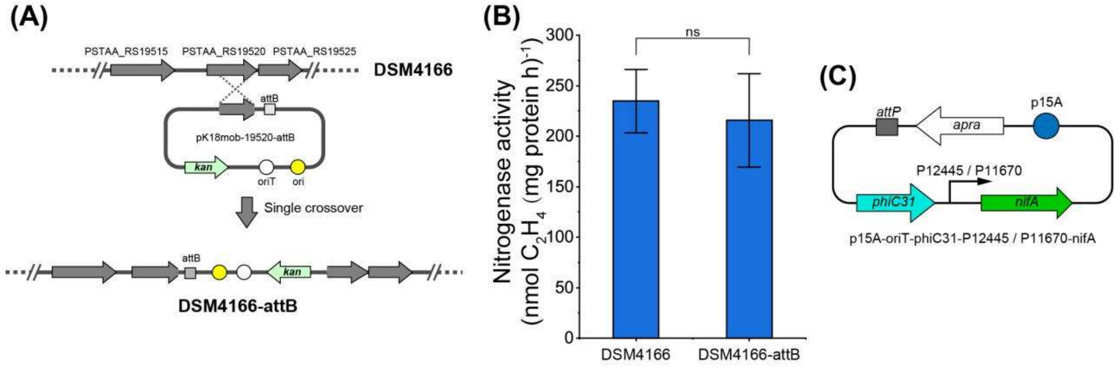

Fig. S4. (**A**) Schematic of construction of DSM4166-attB strain. (**B**) The nitrogenase activity of DSM4166 and DSM4166-attB. (**C**) Schematic of the plasmid containing expression cassette: P12445-nifA or P11670-nifA. Error bars indicate the standard deviations of three replicates (n=3). The P-value cutoff for all the plots is 0.05. * $P<0.05$, ** $P<0.01$, and *** $P<0.001$. ns, not significant. Two-sided Student's t-test was used to compute statistical significance.

Table S3. The sequence of screened endogenous promoters from *P. stutzeri* DSM4166.

**Promoter ID**	**Sequence (5'-3')**
P_{genta}	TGAAGGCACGAACCCAGTTGACATAAGCCTGTTCGGTTCGTAAACTG<br>TAATGCAAGTAGCGTATGCGCTCACGCAACTGGTCCAGAACCTTGAC<br>CGAACGCAGCGGTGGTAACGGCGCAGTGGCGGTTTTCATGGCTTGT<br>TATGACTGTTTTTTTGTACAGTCTATGCCTCGGGCATCCAAGCAGCAA<br>GCGCGTTACGCCGTGGGTCGATGTTTGATGTTATGGAGCAGCAACG
P22180	GCCAAGGCGGCGGGATCGTACCTGCGATCCTGCCGCTGCAGCTGAT<br>CATTTTCTAACCAGCATGATTAAAAAGTGATCGACACGCCGTAAAGAT<br>CTCCGTACACTTGCGCCCAGCACCACAAGCTGCACCA
P17270	CCGGTACGCAGCGCGTTCGCGCGTTGCGTCAGAGGGTGGCTGTCA<br>GCAGGCACGGTCATGCCTCTTTATTAAACACGGGGTCAATCCCCGGC<br>CTGTTCACTCAAGGAGCCCTACC
P19520	GCGCTTCCCTATCCACGACACGGCCGTGCAGCGCACGGCCTGTACG<br>TGTGCTTGAACCGTTATCTCGATACTGTCGGCGCGACCATACGCCGC<br>AGGCCTGCCTTGCTATCACTCGGATTAATACCTAAACAGGAGCATAG<br>CTACGCAAACTGCTGAACCAGAGCCTGCAGAGGCGACAAACGGCAA<br>CAAAACGCGCCAAACCCTTGAGAATCAAGGCGCGTAAGCCTTCTTCG<br>TCGTGTTTCTGACGACATTTTTTAGTCTAAGCCCCTAATATCATTAAG<br>CCTTGGTGCTTGCACTTTGAATTCGGATCGATATCATCAGCCTCGGC<br>CAACCTATTGGGGAAGACCCGCCCCGCCGAATGCCGCTCGTTCCCT<br>TCCGGAAGAGAGCGAGCACCGTAAACGGCAGGGTGTAAAGGACCG<br>GTTCGAAGGACTTCGAACCAACACAACATTTGGAGTGACTCCGTAA
P06545	GGCTGGCGTCCTGAATAGGTTAATGGGCCTGCGAAGAGGCGCATCT<br>TACCGAGCCTGGCGCGGGTGTTCAAAGCCTGGCTACTGGCGCGCCG<br>CAACGGCCGTCACAGCCAGCAGCAGCCACTCGGCGAAGAAATCCAA<br>AATAAAGCTCGCTTTGCTCCAGAAAAACGGTATTGTTACTGGGCTGC<br>TGCTGCATGTGTCACCGTGAATCGCTACATGTTTAGATTTCGATCCAA<br>ATATCGATACGACTCCTATCTCCTTGCACACAGTGATGTTCGGGCCTT<br>TTAGCCCGCACTTTATTCTTCAAAACTTGGTTCAAGGAGACATCAA
P03385	AAAATTATTTCGCCTCAACGCCGCGTCATCCGCGGCCCAGACGCTGT<br>TGTCAGCCGAACGGCAGCGCCGAAAACCCCTCGCGCACCTTGACCT<br>TGCCGAATCCGAACTATCCTTGAGTTCAGGCCATCCAAATAAACGAA

	GCCGGGAAACGGCTGACGGAATTCAGGGTTTCGACCATTAGGTGTT<br>CGGGGGAACTCCACTGGCCGCCACTTCGGCCGTGCTCCAGAGTGCC<br>TGATGACGCGCCTATTCGTGCATCGATCGAGGTGAAGTC
P10035	GGTGAGCTCCTGTTGCGCTGCGCGCTGTGCTGGTCGACCCGCCTCA<br>ACTACAGCATACACCTATACATGGTTATTCGAATGTACAGCTGATGGC<br>GCCCCGTTCGTTGCAGAAAGAGCCGCCACCGTCGGTACCGCAGGGA<br>GCCTGAAGCGCGTATTGACGGCCCGCCAAGGATGCGACACCGCGTT<br>GCTGAGCGTTATTCTGCCGGCAGCCGCCTTCTTGTACAAGAAAAGTC<br>GCAAGATCTTGTACACCACATATTGACTTGTACAAGCCGAAGAATATG<br>ATGGCATCAAGTTGTACAGAAATTCATCTTGTACAAGAGGTTGTCATC
P19680	TGAGGTTTCCTTCACAACAGCGAATACGATGGACCGCGCGATTCAGA<br>GCCTCGCAAGTACCTTTCACAAGGAAATGCCAGTGCCGCTGATCTGT<br>AGTTTTACTACTAAAGAGTATTTCAGGGGGCTGATCCCGGCGCCGTT<br>TTGTAGTAAAACTACAGACGCCCGGCCCTACCCCCGGCACTCGTCCA<br>AGAACATTACACGCACCGGCCCACAAAGCCGGTAGCGAACAGGCGA<br>CCGATTCTGGAAGCCTTTCCGCCATGGAGCAAGCC
P03670	GCGACGACCTTGCGTCTACAGCCCGAGCGACTCGCGACAGGCTGAT<br>GGCTGGTGGCTCTTGCCACCGGCCTTTTTCCGTTATGGAAAGCCAGC<br>CGCGCTGGCTCTTCCTCGGAAGCCACCTACAGGTGGCGCGAACCAT<br>GGGGTTCGCAAGATTTTCTGGCTGCTCCCGTCGGGAGAAGCCAACA<br>AGCAGGTGACCAAGCTGGGGAACGCTG
P20930	ACCAAAAAAAAGCGCCAACGTCGATCATCCGGGGACAGTCTCCGTTG<br>GCGCGCATGTCTCGCGAGGCTTTCCGGGCTTCCTCACCCCGGAAAG<br>AATGTGACGCTGATCTCATATTGATATCAGCGAATCCGCGATCACAA<br>GCGTACTTAGGGAGTACCTTCTTGGAGGTATTTGCCTGCTTGGCACA<br>GCAAACTTGTACAGAATTATAAGTATGTACAATAAATCTATCTTGCAG<br>GCCGCAACCCTGAACGCGATGGCCGGTCTATTTCTGGCATGCGAGC<br>GCCTTTGCCGGTTTCGGCTGTTGCCAGCTCGGTCGCCTGCTAGACT<br>CCCTATTCCGCGCAGACTCACGAGGGATGCCGGATCAACCGAAAAC<br>AACGAGAAAAGGAGTGCATCC
P04125	GGTCGTTCCTGTTTTGTTGTTGTTTGCGAAAGGGTCATGCACAGAACCG<br>CTGTTCTACCGCAAAAGTAGAAAGAAAATCTCTCCGCGTCACGGCCAGA<br>CTGTTAATCGACACGCGACGGTGGGGTGATCCCCTGCCGATCGCCGGC<br>GTGGAGAGACAACCGTGCGGTTTCTCCATGACTGCGTCAGGCGGGTAG<br>CGAACAACGCCACCCTGACTCCGATAACAACAAATTAGGAGCGCCCC

P12415	AAGTTCTCCTTTTGGTCGGTTCGCCGAAGCGCAACTATTATCAGAATT<br>GCGAGCGGGTCAGTAAGGCGATTGAGATAACTGGCAGCTGTATTCG<br>CCTGAAGCCTAAAAAAGAGGCTATGGGCGGGAACCGAAGCGGCGTC<br>ATTTCGTCTTATTGCTTGATGGCGCTTTGCAATCCTGGCTGAAGAAAA<br>ACAAGGAGTTTCCGCT
P07800	CAGAGAATCCTCGTTTCAAAGGTGAAGACTAGCGCGGTGCCGTTTGT<br>CGGCCTAGTCTGAAAAGTAACCGGAGCCGCCCGGATTCCCATGCGT<br>GGCGTTTCTGGCTGAATGTCCATACTGGAGGTTTTGATCGATGAGTA<br>CATTACGGACAGCAGTACCCGTTATTGTC
P09555	TGACACCCCGGTCGCGAAAGGTTATGCGCTAGCCTGGAATGGCCGG<br>ATTGCTTTACCCGTAACACCTGCGGCGCCGAAGTCGAAGCTCGGCG<br>CCGTAGTTTTAGTAGGAATCCCAGCTCACAAAGCCGGGATTTCACTC<br>TGAAAATTTGATGACCAGCAGGAGCATCCGGGTTGTGCCCGGGAAC<br>TATCTGCGCGGTCCGTAGTGGCTTTACCTGAGTCGCTGCTTCAGAAC<br>TTGGGAAGTCATGCTGCGGTTTCCACGCTGGTGGTGTCTCCTTATCG<br>AAGGTGACCAGAC
P10530	TCGAACAAAGGGGCCTTCGGGCCCCTTTGTATTTGGCTAGCCGCCTT<br>GTTTTTTGCCGAGGGTGCCCTCATCTTTGCTGCAAGGGTATAGCCCA<br>TCCGTTTACCGCCGTATGGCCGTCGTAGAGCTGAATTT
P09495	CGCATCAGCAACGGTTCACCCCAGCGAGCCTCTCTGTCAACCCGCT<br>CCCGAATGTTTCACCGTGCCCGTGGGAGCGGCCTTGGCCGCTGAAG<br>CGCTTTTCCAACGTGCAACAGCACACAGCGATCTCCCTTCGCCACTA<br>CTTGGGAAACAGCTTCAGCCATCCCCCTAAAGTTTTCCCCGTTGCGC<br>CCGAAAAGCTGGTCAAGCAAGTCCGCAGTATGGATGCGCTGGCGTA<br>GTGGTGGTGCGGTACCGGGGGCGCGAAAAAGTTCGAATCTGCCCTA<br>AAGCCAACCCAAACGGCGCCGATAAGCTAATCGAATGCGAACTCAAT<br>GGGTGCCTGGGCACAAGCCGGCCCGGAGTCGCAAGCTCAGGCAAC<br>CAAAGTAACTAGCGCCCTTGGAGGCAAACACC
P12505	CGTTTCGGTTCTTCCTGGTGGGCGGCTGGCCCCGGCTGTTGACGCC<br>GATGCCAGCGTGGCCGTCATCGGCTGCTACAGACGGTCGCAGAGG<br>GCCGCAAGGGATGCGCCACTTTACTGCATTAACCCTCGTCTAGACTA<br>ACCTTGAACCTTCGTCCTGTGCCGTCAGTCACTACCGACGAGCCTTT<br>CCCCAGCAAGCCAAGCGGAGCCATAC
P16030	TCACAGTACCAAGCCTGTGCGCAGCGAGCTCCGTATCGATATGCGA<br>CACAGGCAGAAGCGCCTGCTTTACCGCCTTCTCGCTGACTGAAAAAC<br>AACACAGTCAGCCGTGGCTTTGACGATCCGCCGTAGCGGAGAAGCA

	TGTCGAACCACCCTTCAAAAGCGCTGAAATGGCGCCGCCTGGACAG<br>GCAAATTCTATGGCTCCGAGGGGTTGGCATTCCCCGGCGAGTTATC<br>GTAGAGTGTGCCCACTGTGTTTGCATGGGTCGCTGTTGAATCGTGAC<br>CTGGTGCAGTAGATCTTCAGATCCGCTACTCCGCTCGACTCTCTTAC<br>TCCTTGCAACCAGTTCCAGCTTCCTGCTTTGCGGGGGCAATTCTACT<br>TTTGAGTTTCAAGGATACAAAGAC
P05750	GGATCGGCTCCAGTTCAGGGCAAGTCGCCTCGTCTTGGGGCAGGCG<br>GGCGAGGAAATAGCGCGAGGGTACTCGCTCCTGCGACCGGATTGAA<br>CCCGCCCTAGAGCGTGGCGGGCGGCGTCGCATCGAGCTGGTATGC<br>CGCTTGCTTTGGCTGCCTGGCTCTGAACGGAACGGCCCTGGCGAGC<br>GGGGCCGAGGCAGTGAAATAAAGAACATTGCCGATCATCAACGGAC<br>GACTAGACTCAGGTCAGGCTGCGGCCGCTTCGCTGGTCAAGGCATC<br>GAAGCGCCAGGCCCGAACCCGAGAATAAGAAAGCAAACTGGAGGTT<br>TCG
P10410	GTGCCTTGGGCCTTAAGTCGAGAACGGTTCCCAGCTATAGTAGGCC<br>CTTCGCCGAGCCCGGTTGGCGGGCATTGTTACTTATTTTGCAGGGTC<br>GGGCCACAGTCGTGCGGCCCGGCTCCGGTTGAGTTTCCAACACTAA<br>TGTGGAGAGATCACC
P02720	GGGAAATACGCTCCAAATAAAGCGGCTCTGGCGTGAATTATGGCCAA<br>AGATAGCTTTTTATATGGTGAATAACACTGCTTCAGCCTTTTTATACTC<br>CGAATCAATAGTGAATCCGGAAAAGCCTGTTCGAGCGGGCGGGAGG<br>CTGCA
P11845	TCGGAACGCCGAAAGGCGGCCAAACGGACTTCGAGGTCAGCGCAG<br>CCGGTAGGCGCGCCTCGAGCCGTTAACAAGGAGGCATGTGGGAGA<br>CCAGATCCAGTCCCGCAGGCCTTTTTTTATATGAATGAACCCACATTG<br>TCTGGAATGTGGAGATTGGGCGGATTCTCGCCCGAAAACAGCAGGA<br>ATCAAC
P07835	GGTTCATCTCGTTGAAGGAACTTGATACGCCAGGTTCGACTCGCATA<br>GGTATGCTTGGAAGATGGCGAATGCTGAGTCAGGGCGGTGAAGTTG<br>CCAAGTGCCTTTTCGTCGCTGCCACCTAAGCCATCCCCCGGGAACG<br>AAACAGCAGTCTGCAGGAGACTTTCA
P18460	GGCAGTTACGGCGTCGGTGAAGGTTTGCGGGGCAGCCATTGTAGCG<br>GCGCTGCCGTGGCTGCGCTGCGCCATGCTGCAAGAGGCCATCCGG<br>CGAACGGATTGAACTTCGCCGGACCGTCCAACTCCTACCTAGTAAGG<br>GGCGGTTCGCGTGGCCCGGAGAGGGCCCCGCCGTACCGCATTCCG<br>CTGGCACACCAAATGGAGGACATCCC

P18595	GTCGGGCTCCTCGAGTTTGCGCGGAGTATAAAGGCTCGCACCGACC GCGGCAGCGTCAAGCTGCCGACAGCCTACGGATCGCTCGACCGGCT TTTTTTACCTCCTGGCGGAAAACCTGCGAGCATCTGTGAGCCAGGTC ATGTCCAATGAAAAACTGACCTTGGCAGGGGAGCACAATGCTTATAA TCCCCTCCAAGAATATTGACGCCACCTAACTGACGATACTTGCTGAC GAGGCGTCGTTGTAAAATCAAAGCGCTCGGCTATGCTGCGTCTGGAT CCGGTTCGACAGAAGAAACCCGCACAAGATTGCGGGCTCGCCCGGT ACCTTCCGCCTTGCCCTGCCGACTTGGTTACCTATATAAGACGCTGC CGAATTCAAGCGTTGTGACCGCCGCAAAGCAGGCGACCGCAGGAGA GTCTCG
P11670	TTAGCGTTAACTCTCTTCTTGCTGGGCGTGCACAGAATCCCGAATGC GCCGGGTAGGCGCCCTGACTCTCCATCTAGTCGAGCCAGAAGGGGA TTCGCCGCGGTGATGATGGATGCACCAATAGAGTGTCGAGCGTTTGA ACCGCCGAGCCGCGGTGGCAGCGTCAGGGTTCAAGAGGCGGGTCT GGTACAGGCTCGTCCCTTATGACTTGTGAGTCATCGCGGCAGTATAC CATCGAGCCCCCCTCTGGCAGAACGGCATTGCGCATACGACGAACG AACATGTAGTTTCAGGCGATTCGAGTGGGTTACCCTCAAACGACGAT CGATGTCTAACCACGAAGACGGAGTCCAGC
P12445	CACGGCGGCCCGTCGAAGGATTTGTCGAGCAACTGATTCTAGCAGGTCG ATTACCGATCAATTGGCAGTCGGCTGGCCCACCACGCTCGGTGCAGGTC AAAAAACGAGACACCACCCTCACTCAAACAAGAGTTTGACTTCGGTATTC GACTAGTGGCCATCAAACCGTTAGGATTCGGCTGCCCGCCTGAACAATAA TCCGGCGGGCCGGAAGTGGTAAGACGTTTTGTAGCCTAACGAGACTATC GAACAAACCACTCCTGACCTTGACGCCCCGGCCCTAAAGACTAAGGTCT AAAGGCTGAAACAATGATGAAATGTTTTATTGCATGTCGCCGTAAGAGCG ACCTTCAGAGCATCATCCAACGAGGAGAACAAGAA

Table S4. Quality evaluation of samples for RNA-seq.

**Sample name**	**Concentration (ng μL^{-1})**	**Total (μg)**	**OD260/280**	**OD260/230**	**23S/16S**	**RIN value**
LB-10h	1876	60.032	2.066	1.712	1.8	9.7
LB-14h	1992	63.744	2.045	2.274	1.9	9.3
PMM-28h	742	23.744	2.05	1.364	1.6	10
PMM-38h	964	30.848	2.008	1.01	2.1	8.9

Table S5. Oligonucleotides for cloning of screened endogenous promoter regions from *P. stutzeri* DSM4166.

**Primer name**	**Sequence (5’-3’)**	**PCR product**
pBBR1-1	CCTCATAAAGGCCAAGAAGGGCGGAAAGTCCAAATTGT AAGCTAGCCATATGGCTTACCG	pBBR1 vector
pBBR1--2	ATCTGCATAAAAACTGTTGTAAT	
firefly-1	ATGGAAGACGCCAAAAACAT	*firefly*
firefly-2	TTACAATTTGGACTTTCCGCC	
02720-1	GGCAGAATGCTTAATGAATTACAACAGTTTTTATGCAGA TGGGAAATACGCTCCAAATAA	P02720
02720-2	ATGGCGCCGGGCCTTTCTTTATGTTTTTGGCGTCTTCCA TTGCAGCCTCCCGCC	
03385-1	GGCAGAATGCTTAATGAATTACAACAGTTTTTATGCAGA TAAAATTATTTCGCCTCAACGC	P03385
03385-2	ATGGCGCCGGGCCTTTCTTTATGTTTTTGGCGTCTTCCA TGACTTCACCTCGATCG	
03670-1	GGCAGAATGCTTAATGAATTACAACAGTTTTTATGCAGA TAGCTCAAGTAAGCGACGACC	P03670
03670-2	ATGGCGCCGGGCCTTTCTTTATGTTTTTGGCGTCTTCCA TCAGCGTTCCCCAGCTT	
04125-1	GGCAGAATGCTTAATGAATTACAACAGTTTTTATGCAGA TTGGTCGTTCCTGTTTTGTTGT	P04125
04125-2	ATGGCGCCGGGCCTTTCTTTATGTTTTTGGCGTCTTCCA TGGGGCGCTCCTAATTTG	
05750-1	GGCAGAATGCTTAATGAATTACAACAGTTTTTATGCAGA TGGATCGGCTCCAGTTCAGG	P05750

05750-2	ATGGCGCCGGGCCTTTCTTTATGTTTTTGGCGTCTTCCATCGAAACCTCCAGTTTGC	
06545-1	GGCAGAATGCTTAATGAATTACAACAGTTTTTATGCAGATGGCTGGCGTCCTGAATAG	P06545
06545-2	ATGGCGCCGGGCCTTTCTTTATGTTTTTGGCGTCTTCCATTTGATGTCTCCTTGAACC	
07800-1	GGCAGAATGCTTAATGAATTACAACAGTTTTTATGCAGATCAGAGAATCCTCGTTTCAAA	P07800
07800-2	ATGGCGCCGGGCCTTTCTTTATGTTTTTGGCGTCTTCCATGACAATAACGGGTACTG	
07835-1	GGCAGAATGCTTAATGAATTACAACAGTTTTTATGCAGATATGGTTCATCTCGTTGAAGG	P07835
07835-2	ATGGCGCCGGGCCTTTCTTTATGTTTTTGGCGTCTTCCATTGAAAGTCTCCTGCAGA	
09495-1	GGCAGAATGCTTAATGAATTACAACAGTTTTTATGCAGATCGCATCAGCAACGGTTCAC	P09495
09495-2	ATGGCGCCGGGCCTTTCTTTATGTTTTTGGCGTCTTCCATGGTGTTTGCCTCCAAG	
09555-1	GGCAGAATGCTTAATGAATTACAACAGTTTTTATGCAGATTACCTGATGACACCCCGGTC	P09555
09555-2	ATGGCGCCGGGCCTTTCTTTATGTTTTTGGCGTCTTCCATGTCTGGTCACCTTCGATA	
10035-1	GGCAGAATGCTTAATGAATTACAACAGTTTTTATGCAGATTTCATGGTGAGCTCCTGTTG	P10035
10035-2	ATGGCGCCGGGCCTTTCTTTATGTTTTTGGCGTCTTCCATGATGACAACCTCTTGTAC	
10410-1	GGCAGAATGCTTAATGAATTACAACAGTTTTTATGCAGATGCAGTTCGTTTCATGTGCC	P10410

10410-2	ATGGCGCCGGGCCTTTCTTTATGTTTTTGGCGTCTTCCA<br>TGGTGATCTCTCCACATTAGT	
10530-1	GGCAGAATGCTTAATGAATTACAACAGTTTTTATGCAGA<br>TGATCGAACAAAGGGGCCTT	P10530
10530-2	ATGGCGCCGGGCCTTTCTTTATGTTTTTGGCGTCTTCCA<br>TAAATTCAGCTCTACGAC	
11670-1	GGCAGAATGCTTAATGAATTACAACAGTTTTTATGCAGA<br>TGGACATTTAGCGTTAACTCTC	P11670
11670-2	ATGGCGCCGGGCCTTTCTTTATGTTTTTGGCGTCTTCCA<br>TGCTGGACTCCGTCTT	
11845-1	GGCAGAATGCTTAATGAATTACAACAGTTTTTATGCAGA<br>TTTGGCTGGATGATCGGAAC	P11845
11845-2	ATGGCGCCGGGCCTTTCTTTATGTTTTTGGCGTCTTCCA<br>TGTTGATTCCTGCTGTTTT	
12415-1	ATGGCGCCGGGCCTTTCTTTATGTTTTTGGCGTCTTCCA<br>TAGCGGAAACTCCTTGTT	P12415
12415-2	GGCAGAATGCTTAATGAATTACAACAGTTTTTATGCAGA<br>TAAGTTCTCCTTTTGGTCGG	
12445-1	GGCAGAATGCTTAATGAATTACAACAGTTTTTATGCAGA<br>TCATTCAGGAAGCATCACGGC	P12445
12445-2	ATGGCGCCGGGCCTTTCTTTATGTTTTTGGCGTCTTCCA<br>TTTCTTGTTCTCCTCGTTGG	
12505-1	GGCAGAATGCTTAATGAATTACAACAGTTTTTATGCAGA<br>TCGTTTCGGTTCTTCCTGG	P12505
12505-2	ATGGCGCCGGGCCTTTCTTTATGTTTTTGGCGTCTTCCA<br>TGTATGGCTCCGCTTG	
16030-1	GGCAGAATGCTTAATGAATTACAACAGTTTTTATGCAGA<br>TCCATCACAGTACCAAGCC	P16030

16030-2	ATGGCGCCGGGCCTTTCTTTATGTTTTTGGCGTCTTCCA TGTCTTTGTATCCTTGAAAC	
17270-1	GGCAGAATGCTTAATGAATTACAACAGTTTTTATGCAGA TTCGGAATGACCGGTACGCAG	P17270
17270-2	ATGGCGCCGGGCCTTTCTTTATGTTTTTGGCGTCTTCCA TGGTAGGGCTCCTTGAG	
18460-1	GGCAGAATGCTTAATGAATTACAACAGTTTTTATGCAGA TGGCAGTTACGGCGTCGG	P18460
18460-2	ATGGCGCCGGGCCTTTCTTTATGTTTTTGGCGTCTTCCA TGGGATGTCCTCCATTTG	
18595-1	GGCAGAATGCTTAATGAATTACAACAGTTTTTATGCAGA TGTCGGGCTCCTCGAGTTTG	P18595
18595-2	ATGGCGCCGGGCCTTTCTTTATGTTTTTGGCGTCTTCCA TCGAGACTCTCCTGCGG	
19520-1	GGCAGAATGCTTAATGAATTACAACAGTTTTTATGCAGA TGCGCTTCCCTATCCACGAC	P19520
19520-2	ATGGCGCCGGGCCTTTCTTTATGTTTTTGGCGTCTTCCA TTTACGGAGTCACTCCAAA	
19680-1	GGCAGAATGCTTAATGAATTACAACAGTTTTTATGCAGA TTGAGGTTTCCTTCACAACAG	P19680
19680-2	ATGGCGCCGGGCCTTTCTTTATGTTTTTGGCGTCTTCCA TGGCTTGCTCCATGGCG	
20930-1	GGCAGAATGCTTAATGAATTACAACAGTTTTTATGCAGA TACCAAAAAAAAGCGCCAACG	P20930
20930-2	ATGGCGCCGGGCCTTTCTTTATGTTTTTGGCGTCTTCCA TGGATGCACTCCTTTTCTC	
22180-1	GGCAGAATGCTTAATGAATTACAACAGTTTTTATGCAGA TGCCAAGGCGGCGGGATCG	P22180

22180-2	ATGGCGCCGGGCCTTTCTTTATGTTTTTGGCGTCTTCCA TTGGTGCAGCTTGTGGTGC	
P_{genta}-1	GGCAGAATGCTTAATGAATTACAACAGTTTTTATGCAGA TTGAAGGCACGAACCCAGTTG	P_{genta}
P_{genta}-2	ATGGCGCCGGGCCTTTCTTTATGTTTTTGGCGTCTTCCA TCGTTGCTGCTCCATAAC	

36

Table S6. Oligonucleotides used for construction of DSM4166-attB strain.

**Primer name**	**sequence (5'-3')**	**PCR product**
pK18mob-1	GCCAGGGCGTGCCCTTGGGCTCCCCGGGCGCGTACTCCACCTCACCCATCTGGTCCATCATGATGAATTCGTAATCATGGTCATAG	pK18mob vector
pK18mob-2	GGCACTGGCCGTCGTTTTAC	
19520-1	GTTTTCCCAGTCACGACGTTGTAAAACGACGGCCAGTGCCATGGCAACGAAACTCTGG	PSTAA_19520
19520-2	GTGGAGTACGCGCCCGGGGAGCCCAAGGGCACGCCCTGGCACCCGCACCGCGGCTTCGAGACCGTCCCTTGGAGACGGTGTAAGAC	
19520-check-F	AAACGATGTCGCCGATCTTC	Crossover-L colony PCR check
lac-seq	AGGCACCCCAGGCTTTACAC	
pK18-seq	CGGGCCTCTTCGCTATTAC	Crossover-R colony PCR check
19520-check-R	TAGAGGGGGCGAGACATAAG	

Table S7. Oligonucleotides used for construction of DSM4166-P12445-nifA and DSM4166-P11670-nifA strains.

Primer name	sequence (5’-3’)	PCR product
p15A-1	AGATCCGAAAACCCCAAGTT	p15A vector
p15A-2	AGATCCTTTCTCCTCTTTAGATC	
nifA-1	ATGAACGCCACATTTGCCGA	*nifA*
nifA-2	TGGAGATCCTTAAGATCCGTAACTTGGGGTTTTCGGATCT TCAGATCTTGCGCATATGAA	
P12445-F with p15A HA	AGAAAAGAATTCAAAAGATCTAAAGAGGAGAAAGGATCTC ATTCAGGAAGCATCACGGC	P12445 for P12445-nifA
P12445-R with nifA HA	GGGTCGGGGCGACGGCGCGTTCGGCAAATGTGGCGTTC ATTTCTTGTTCTCCTCGTTGG	
P11670-F with p15A HA	AGAAAAGAATTCAAAAGATCTAAAGAGGAGAAAGGATCTT TAGCGTTAACTCTCTTCTTGC	P11670 for P11670-nifA
P11670-R with nifA HA	GGGTCGGGGCGACGGCGCGTTCGGCAAATGTGGCGTTC ATGCTGGACTCCGTCTTCGTG	
19520-check-F	AAACGATGTCGCCGATCTTC	*attL* site colony PCR check
nifA-5out	TGTTGCCGTGCTTGAGGATG	
lac-seq	AGGCACCCCAGGCTTTACAC	*attR* site colony PCR check
nifA-3out	GTACGCGAACTGGAAAACTG	

Table S8. Oligonucleotides for quantitative real-time PCR.

Primer name	Sequence (5’-3’)
GAPDH-F	CAATGGGTATGGGCGTATCG
GAPDH-R	AGGTGGCTGAGGTCGTTGAT
nifA-qRT-F	CAAGTGGAACAGCACCGT
nifA-qRT-R	GGCGAGTTGTAGTGGATGG
nifB-qRT-F	GACCATCACCATCAACTGC
nifB-qRT-R	TTGACCTTCACCAGGATGC
nifD-qRT-F	CGCTTGAGCAATCGAAGAA
nifD-qRT-R	AGACCACACCCTTCGAGC
nifE-qRT-F	TCAAGTCCTGGTCGGTGAT
nifE-qRT-R	CCTCCTCGGTGGATTTCTT
nifF-qRT-F	CCGATTGCGAGAACGAAAG
nifF-qRT-R	CCCATCGCATCGAGAAACTC
nifH-qRT-F	GGATGACCTGGACTTCGTCT
nifH-qRT-R	GCATACATCGCCATCATCTC
nifK-qRT-F	AACCCTGGCAGTTGGAAA
nifK-qRT-R	TGACCTTCATCAGGAACTCG
nifM-qRT-F	ATCACCGATACAGAGGTCAGC
nifM-qRT-R	GGAAATCCTCGTTGACGGT
nifN-qRT-F	TTCGCCAAGGTGTTCTTCG

nifN-qRT-R	GATGCTGCTTGTCGCAAATG
nifQ-qRT-F	AGCATCTATGGCGCGACCT
nifQ-qRT-R	TCTTCCAGCGCATGTCCTG
nifS-qRT-F	TCAAGGTGGACAAGAAGGG
nifS-qRT-R	CGCATCGCTATGGAACAT
nifT-qRT-F	GATCAGCCGTAACAAGAATG
nifU-qRT-F	TGTCGCTGAAGGTCGATCC
nifU-qRT-R	TGATCTTCAGCGCCTCGTC
nifV-qRT-F	GATACGGTGGGAGTAATGGAG
nifV-qRT-R	GCACTTCCAGCTCCAGATC
nifW-qRT-F	CTGAACTTCTTCGGCGTG
nifW-qRT-R	TGACACAGGTAGTCGTGATAGC
nifX-qRT-F	CGTTCGTGTTCTACGGCATCG
nifX-qRT-R	TTGTCCTCGTTGCCGTCCT
nifY-qRT-F	AGCTGAACGGCGTGGATAC
nifY-qRT-R	GTTGCGCAGGCGGTTTAG
nifZ-qRT-F	TGGATACCCAACCTGTTCG
nifZ-qRT-R	AATAGACGTGGTACTGGGTGC
rnfA-qRT-F	GGTCATGGTGATCTTCGCC
rnfA-qRT-R	AAAGCCATCGCCAGCAAC
rnfB-qRT-F	ATCCATTGCGTGTTGAGC

rnfB-qRT-R	AGTGGTCCAGCGTCAGTGT
rnfC-qRT-F	CCTACAGCATCGTCATCGCC
rnfC-qRT-R	TGGCGGATCAGCTGCTTG
rnfD-qRT-F	CTGTATCTGTTCGGCTGGCC
rnfD-qRT-R	CGTCAGTGCCAACAGCCAG
rnfE-qRT-F	CATTGGCTGGAAATCACCG
rnfE-qRT-R	TTGAACGCCAGCACGAAG
rnfG-qRT-F	CTACGACAACGATCCGCTG
rnfG-qRT-R	GACGGTGCTGATCTGGAAG
rnfH-qRT-F	ACGGCAAGTTCGTCAAAC
rnfH-qRT-R	CGTCATCGTCGTCTTCATC

44

Table S9. Nitrogenase activity of DSM4166, DSM4166-P11670-nifA, DSM4166-P12445-nifA.

Strains	C_2H_4 yield (nmol)	Protein (mg)	Nitrogenase activity (nmol C_2H_4/mg protein/h)	Nitrogenase activity mean	SD
	36.420	0.046	198.713		
DSM4166	48.073	0.047	256.980	234.657	31.431
	48.328	0.049	248.278		
	81.800	0.052	391.352		
DSM4166-P11670-nifA	97.459	0.050	487.197	431.281	49.883
	100.974	0.060	415.293		
	183.665	0.052	882.071		
DSM4166-P12445-nifA	199.990	0.053	945.076	954.687	77.868
	242.804	0.059	1036.915		

Table S10. Quantitative real-time PCR (qRT-PCR) analysis of *nif* genes in DSM4166 and DSM4166-P12445-nifA strains.

Genes	Strains	Relative quantification (RQ*)			RQ mean	SD
nifH	DSM4166	1.521	0.704	0.774	1.000	0.453
	DSM4166-P12445-nifA	83.774	121.889	70.747	92.137	26.577
nifD	DSM4166	1.512	0.688	0.800	1.000	0.447
	DSM4166-P12445-nifA	91.379	112.729	70.443	91.517	21.143
nifK	DSM4166	1.520	0.719	0.761	1.000	0.451
	DSM4166-P12445-nifA	131.673	152.841	76.567	120.360	39.375
nifT	DSM4166	1.411	0.864	0.725	1.000	0.362
	DSM4166-P12445-nifA	116.534	172.091	94.982	127.869	39.785
nifY	DSM4166	1.490	0.712	0.798	1.000	0.427
	DSM4166-P12445-nifA	118.705	143.046	111.488	124.413	16.535
nifE	DSM4166	1.627	0.642	0.731	1.000	0.545
	DSM4166-P12445-nifA	62.007	41.976	47.827	50.603	10.300
nifN	DSM4166	1.608	0.634	0.757	1.000	0.530
	DSM4166-P12445-nifA	41.962	42.482	30.402	38.282	6.829
nifX	DSM4166	1.623	0.641	0.736	1.000	0.542
	DSM4166-P12445-nifA	93.974	75.231	87.539	85.581	9.524
nifB	DSM4166	1.442	0.712	0.846	1.000	0.388
	DSM4166-P12445-nifA	69.933	105.061	47.762	74.252	28.893

nifQ	DSM4166	1.284	0.791	0.925	1.000	0.255
	DSM4166-P12445-nifA	87.466	93.915	83.240	88.207	5.376
nifU	DSM4166	1.610	0.637	0.753	1.000	0.532
	DSM4166-P12445-nifA	44.794	50.469	31.657	42.307	9.650
nifS	DSM4166	1.590	0.632	0.778	1.000	0.516
	DSM4166-P12445-nifA	28.446	15.967	17.529	20.647	6.799
nifV	DSM4166	1.589	0.661	0.750	1.000	0.512
	DSM4166-P12445-nifA	58.775	39.128	50.991	49.631	9.894
nifW	DSM4166	1.570	0.667	0.764	1.000	0.496
	DSM4166-P12445-nifA	59.162	69.138	68.035	65.445	5.469
nifZ	DSM4166	1.480	0.675	0.845	1.000	0.424
	DSM4166-P12445-nifA	27.134	30.292	23.372	26.933	3.464
nifM	DSM4166	1.506	0.677	0.817	1.000	0.444
	DSM4166-P12445-nifA	59.802	50.525	57.063	55.797	4.766
nifF	DSM4166	1.506	0.650	0.845	1.000	0.449
	DSM4166-P12445-nifA	38.330	49.814	26.934	38.359	11.440
rnfA	DSM4166	1.500	0.705	0.796	1.000	0.435
	DSM4166-P12445-nifA	48.482	29.354	34.645	37.494	9.877
rnfB	DSM4166	1.588	0.628	0.784	1.000	0.516
	DSM4166-P12445-nifA	30.266	24.332	22.576	25.725	4.030
rnfC	DSM4166	1.540	0.608	0.852	1.000	0.483

	DSM4166-P12445-nifA	24.818	34.969	20.661	26.816	7.360
rnfD	DSM4166	1.562	0.629	0.809	1.000	0.495
	DSM4166-P12445-nifA	50.756	44.312	46.634	47.234	3.264
rnfG	DSM4166	1.356	0.683	0.961	1.000	0.338
	DSM4166-P12445-nifA	16.900	17.784	17.378	17.354	0.442
rnfE	DSM4166	1.352	0.760	0.887	1.000	0.312
	DSM4166-P12445-nifA	48.717	66.485	51.510	55.571	9.555
rnfH	DSM4166	1.356	0.751	0.893	1.000	0.316
	DSM4166-P12445-nifA	53.763	63.742	50.193	55.899	7.023

*RQ was calculated using $2^{-\Delta\Delta Ct}$ method. The expression level of *nif* genes in DSM4166 wild-type strain were defined as 1. The RQ of *nif* genes in DSM4166-P12445-nifA strain represented the expression levels compared to the wild-type strain.

Table S11. The concentration of extracellular ammonium produced by DSM4166 and *nifA* overexpression strains.

Strains	NH_4^+ concentration (μM)			Average	STDEV
DSM4166	3.29	20.17	18.42	13.96	9.28
DSM4166-P11670-nifA	20.98	19.43	25.62	22.01	3.22
DSM4166-P12445-nifA	408.63	321.23	347.39	359.08	44.86
